# Supplementary figures and images for: In silico prediction of ARB resistance: A first step in creating personalized ARB therapy
Source: PLoS Comput Biol. 2020 Nov 25;16(11):e1007719. doi: 10.1371/journal.pcbi.1007719 (PMC7725353; doi:10.1371/journal.pcbi.1007719)

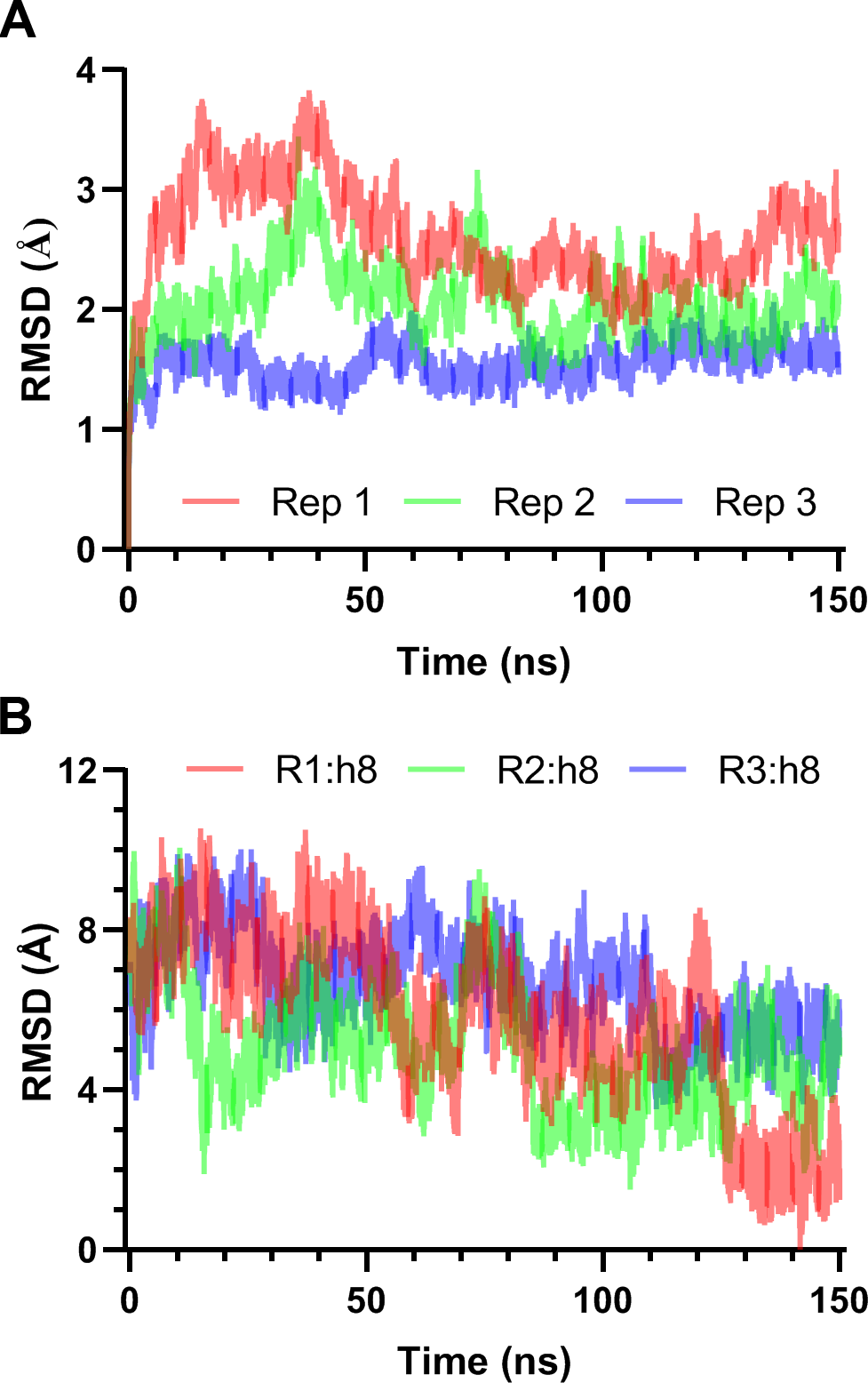

Supplement: S1 Fig — A, RMSD of each model indicates that the empty AT1R models are stable; B, however, helix 8 is mobile when global movement is minimal. The apo-AT1R model served as the reference for helix 8 mobility, and it is the only frame that orientates helix 8 as an extension of helix 7 (0 RMSD in replica 1 red line R1:h8). (TIF) [file pcbi.1007719.s001.tif]

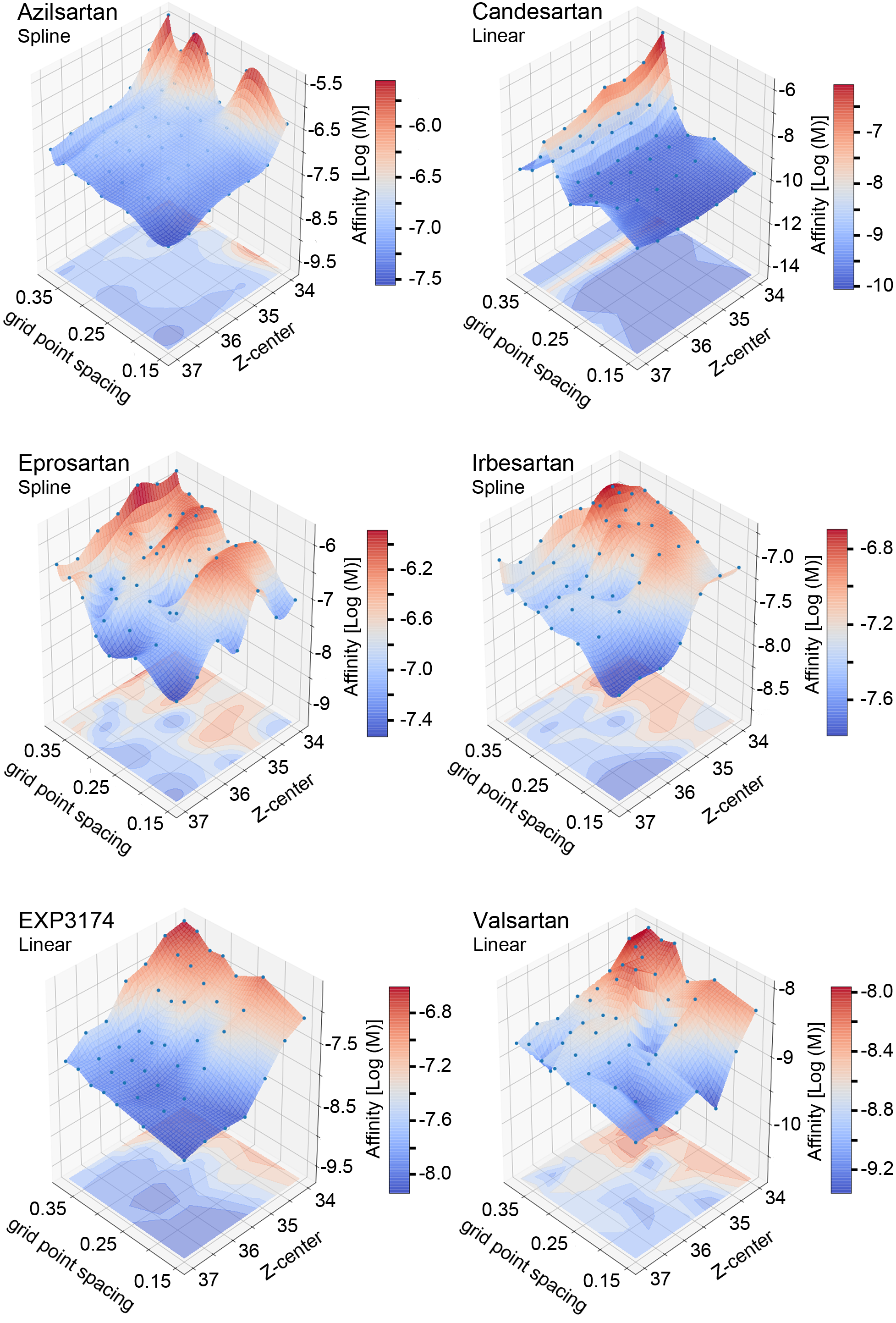

Supplement: S3 Fig — 3D fitting to optimize AutoDock 4.2 grid point spacing and grid box Z-center for ARB binding to the apo-AT1R model. The fit providing the optimal parameters is listed below the ARB. The seven-by-seven table best-modeled Telmisartan; thus, Telmisartan is not shown. (TIF) [file pcbi.1007719.s003.tif]

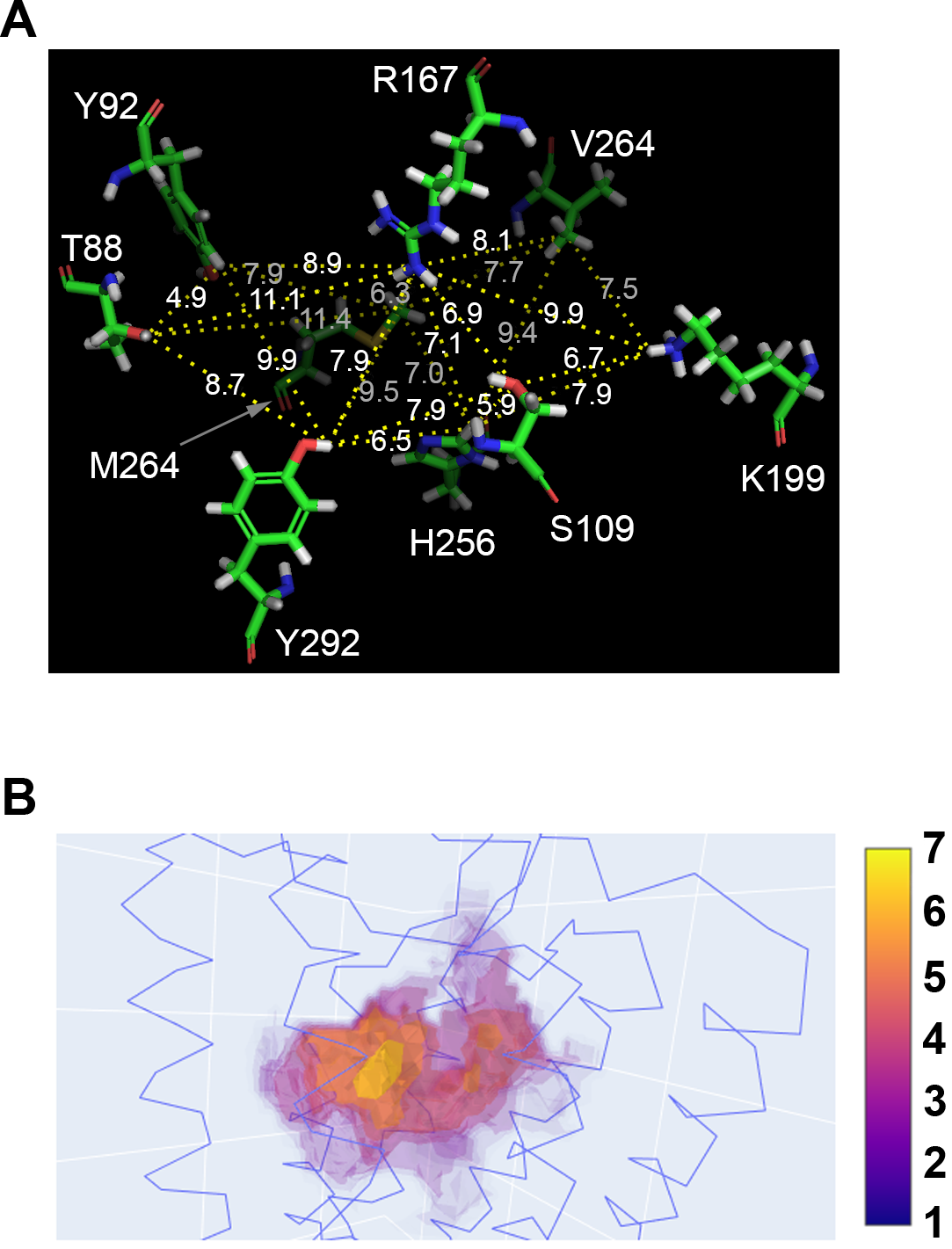

Supplement: S4 Fig — A, Residues lining the binding pocket were identified in the structure, and the distances between them determined to create a search space for water. B, The same residues were used to create a breadth-first search of the space identified by the intersection of the ligand-binding pocket centroid sphere and at least one 8 Å sphere from each residue atom listed in the methods. The color scale represents the area within the centroid sphere overlapping with one to seven of the residue spheres, and the purple lines are a backbone trace of the AT1R. The image displayed matches the frame used to create the apo-AT1R model. (TIF) [file pcbi.1007719.s004.tif]
