# Supplementary material for: In silico prediction of ARB resistance: A first step in creating personalized ARB therapy
Source: PLoS Comput Biol. 2020 Nov 25;16(11):e1007719. doi: 10.1371/journal.pcbi.1007719 (PMC7725353; doi:10.1371/journal.pcbi.1007719)
Supplement: S2 Fig — The apo-AT1R model (grey) is aligned to the, A, inactive A2AR, B, active AT1R, and, C, active μOR crystal structures (gold). The activation motifs are expanded in boxes with the residues labeled by single amino acid letter and structure shown as sticks colored by atom type, with the carbons grey and gold for the apo-AT1R model and comparative crystal structure, respectively. (PDF) [file pcbi.1007719.s002.pdf]

S2A

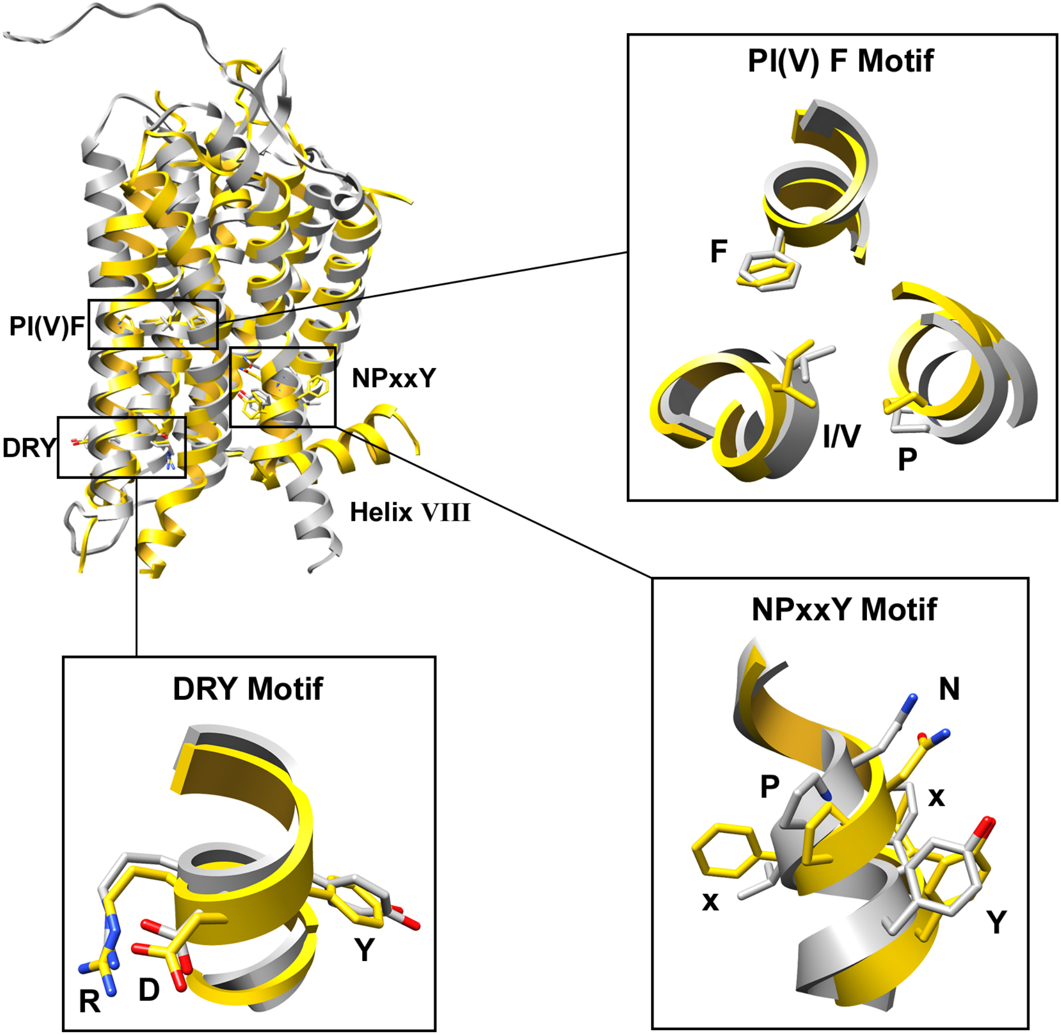

Comparison of the apo-AT_1R model (grey) to inactive $A_{2A}R$ (PDB: 3EML, gold)

S2B

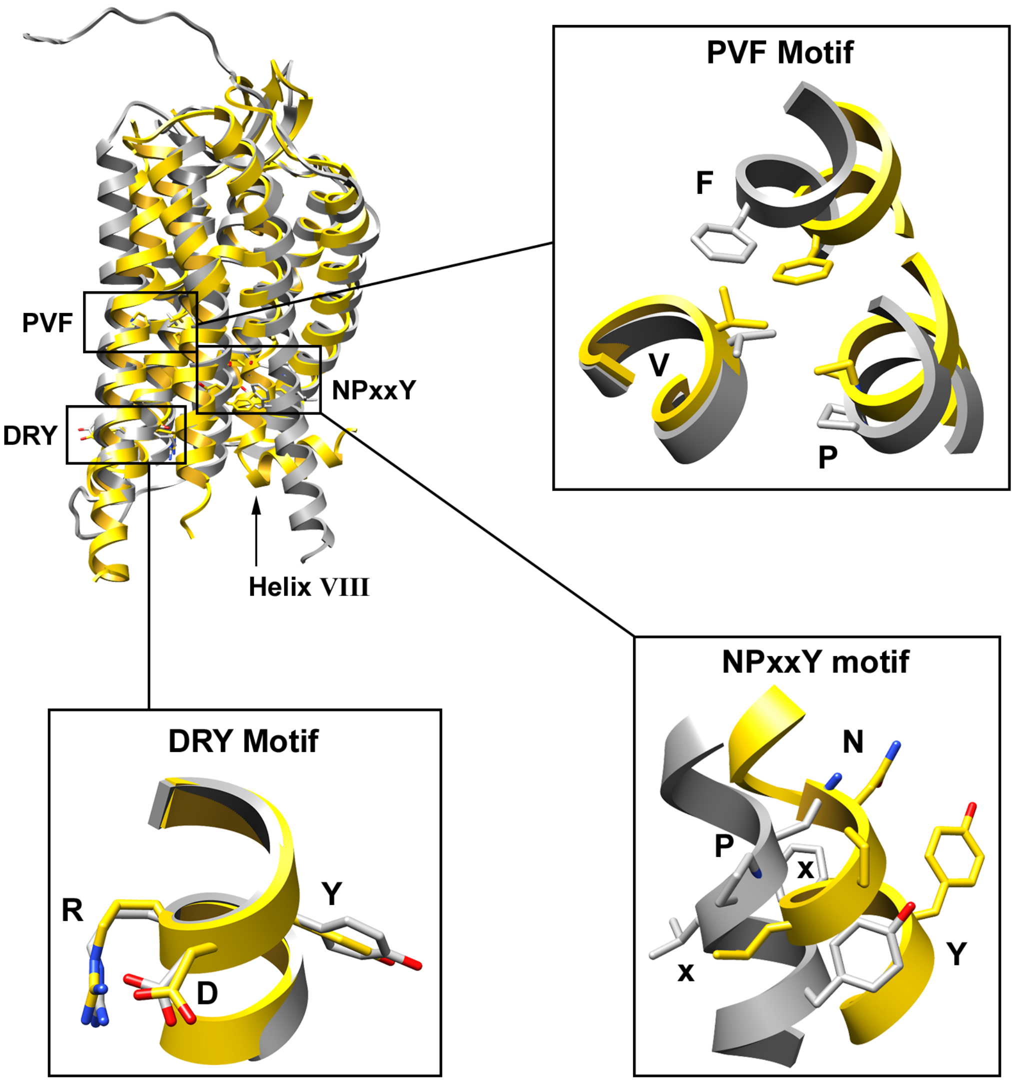

Comparison of the apo-AT_1R model (grey) to active AT_1R (PDB: 6DO1, gold)

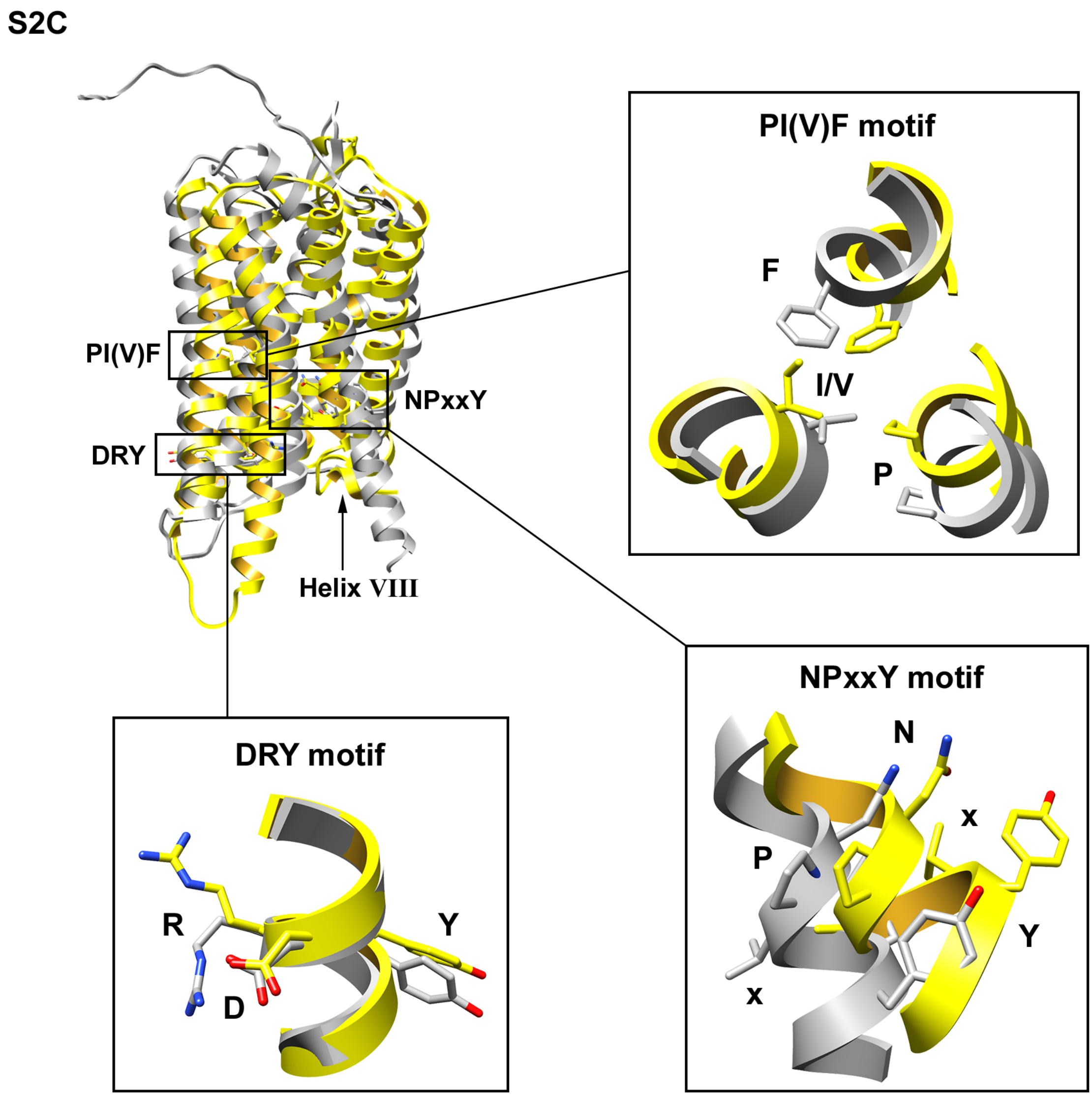

Comparison of the apo-AT$_1$R model (grey) to active μOR (PDB:5C1M, gold)
