# Supplementary material for: In silico prediction of ARB resistance: A first step in creating personalized ARB therapy
Source: PLoS Comput Biol. 2020 Nov 25;16(11):e1007719. doi: 10.1371/journal.pcbi.1007719 (PMC7725353; doi:10.1371/journal.pcbi.1007719)
Supplement: S1 Text — The script is written in MS Word and cannot be cut and pasted to run due to different handling of fonts. Notes to help the user are in red boxes on the left-hand side of the text. (PDF) [file pcbi.1007719.s005.pdf]

Supplemental Methods: Scripts utilized to optimize AutoDock parameters.

```
import scipy as sp
import numpy as np
import pandas as pd
import ggplot
from ggplot import *
from mpl_toolkits.mplot3d import Axes3D
import matplotlib.pyplot as plt
from matplotlib import cm
import plotly.plotly as ply
import plotly.graph_objs as pob
```

CELL BREAK

```
dataFileName="Val-nM.csv"
bindingData=pd.read_csv(dataFileName,header=None,
                    names=["GridSpacing","Zcenter","Affinity"])
bindingData["GridSpacing"]=bindingData["GridSpacing"]*.001
bindingData["LogAffinity"]=np.log10(bindingData["Affinity"]*1E-9)
bindingData[1:10]
```

Note: 7x7 data arranged in columns with nM affinity and saved as ARB-nM.csv where ARB is the ARB examined, Valsartan in this instance

CELL BREAK

```
xi=np.linespace(0.150,.375,(int)(np.floor(1+(.375-.150)/.001)))
yi=np.linespace(34,37,(int)(np.floor(1+(37-34)/.01)))
print xi.shape
print yi.shape
```

Note: generates arrays of grid values along x and y

CELL BREAK

```
grid_x,grid_y=np.meshgrid(xi,yi)
print grid_x.shape
print grid_y.shape
```

Note: generates 2d grids of gridvalues for x and y

CELL BREAK

```
expVal=np.log10(3.47*(10**(-9)))

for interpType in ['nearest','linear','cubic']:
   points=np.array(bindingData[["GridSpacing"."Zcenter"]])
   values=np.array(bindingData["LogAffinity"])
   grid_interp=sp.interpolate.griddata(points,values,(grid_x,grid_y),method=interpType)

   print "#################",
   print interpType,
   print "#################"
```

```
minInd=np.argmin((grid_interp.flatten()))
minX=grid_x.flatten()[minInd]
minY=grid_y.flatten()[minInd]
minInterp=grid_interp.flatten()[minInd]

minExpInd=np.argmin(abs(grid_interp.flatten()-expVal))
minExpX=grid_x.flatten()[minExpInd]
minExpY=grid_y.flatten()[minExpInd]
minExpInterp=grid_interp.flatten()[minExpInd]

print "Exp Value:"+str(expVal)
print "min X: "+str(minX)
print "min Y: "+str(minY)
print "min Z: "+str(minInterp)
print " "
print "Exp X: "+str(minExpX)
print "Exp Y: "+str(minExpY)
print "Exp Z: "+str(minExpInterp)

contourPlot=plt.imshow(grid_interp, cmap=cm.coolwarm)

zmax=np.max(grid_interp.flatten())
zrange=np.max(grid_interp.flatten())-np.min(grid_interp.flatten())
zmin=np.min(grid_interp.flatten())-zrange

fig = plt.figure(figsize=(12,12))
ax = fig.gca(projection='3d')

cset = ax.contourf(grid_x,grid_y,grid_interp,
               zdir='z', offset=zmin, cmap=cm.coolwarm,
               alpha=.5)

surf = ax.plot_surface(grid_x,grid_y,grid_interp, cmap=cm.coolwarm,
                linewidth=0, antialiased=False,
                alpha=.75)

scatterpoints= ax.scatter3D(np.array(bindingData["GridSpacing"]),
    np.array(bindingData["Zcenter"]),
    np.array(bindingData["LogAffinity"]),alpha=1)

ax.set_zlim(zmin,zmax)

ax.view_init(30,135)

fig.colorbar(surf, shrink=0.5, aspect=10)
```

This will produce three of the graphs (nearest, linear, and cubic) with the minimum affinity values and those that match, or are closest to, the expected value.

```
    plt.show ()

CELL BREAK

#create interpolation
interpFit=sp.interpolate.LSQBivariateSpline(
    np.array(bindingData["GridSpacing"]),
    np.array(bindingData["Zcenter"]),
    np.array(bindingData["LogAffinity"]),
    np.array(bindingData["GridSpacing"].unique()),
    np.array(bindingData["Zcenter"].unique()))
grid_interp=interpFit.ev(grid_x,grid_y)
print grid_interp.shape
contourPlot=plt.imshow(grid_interp, cmap=cm.coolwarm)
```

This creates the 2D spline graph

```
CELL BREAK

zmax=np.max(grid_interp.flatten())
zrange=np.max(grid_interp.flatten())-np.min(grid_interp.flatten())
zmin=np.min(grid_interp.flatten())-zrange

fig = plt.figure()
ax = fig.gca(projection='3d')

cset = ax.contourf(grid_x,grid_y,grid_interp,
                   zdir='z', offset=zmin, cmap=cm.coolwarm,
                   alpha=.5

surf = ax.plot_surface(grid_ x,grid_y,grid_interp, cmap=cm.coolwarm,
                   linewidth=0, antialiassed=False,
                   alpha=.75

scatterpoints= ax.scatter3D(np.array(bindingData["GridSpacing"]),
                   np.array(bindingData["Zcenter"],
                   np.array(bindingData["LogAffinity:]),alpha=1

ax,set_zlim(zmin,zmax)

ax.view_init(30,135)

fig.colorbar(surf, shrink=0.5, aspect=10)

plt.shopw()

CELL BREAK

expVal=np.log10(3.47*(10**(-9)))
```

This creates the 3D spline graph

```
minInd=np.argmin((grid_interp.flatten()))
minX=grid_x.flatten()[minInd]
minY=grid_y.flatten()[minInd]
minInterp=grid_interp.flatten()[minInd]

minExpInd=np.argmin(abs(grid_interp.flatten()-expVal))
minExpX= grid_x.flatten()[minExpInd]
minExpY= grid_y.flatten()[minExpInd]
minExpInterp=grid_interp.flatten()[minExpInd]

print “min X: “+str(minX)
print “min Y: “+str(minY)
print “min Z: “+str(minInterp)
print “ “
print “Exp X: “+str(minExpX)
print “Exp Y: “+str(minExpY)
print “Exp Z: “+str(minExpInterp)
```

This creates the minimum and expected values from the spline.
