# Supplementary material for: In silico prediction of ARB resistance: A first step in creating personalized ARB therapy
Source: PLoS Comput Biol. 2020 Nov 25;16(11):e1007719. doi: 10.1371/journal.pcbi.1007719 (PMC7725353; doi:10.1371/journal.pcbi.1007719)
Supplement: S2 Text — The script is written in MS Word and cannot be cut and pasted to run due to different handling of fonts. Notes to help the user are in red boxes on the left-hand side of the text. (PDF) [file pcbi.1007719.s006.pdf]

Supplemental Methods: Script utilized to calculate ligand binding pocket volume

```
import numpy as np
import scipy as sp
import pandas as pd
import pytraj as pt
import miniball
import gridData
import itertools
import os
import tqdm
import gc
import copy
import time
import plotly as ply
from plotly import graph_objs as go
ply.io.renderers.default="notebook"
import importlib
import ipywidgets as widgets
from ipywidgets import interact, interact_manual
import matplotlib
from matplotlib import pyplot as ply
```

CELL BREAK

Define rendering and pocket volume calculation functions

CELL BREAK

```
def get_traj_pocket_centerCOM_table(traj,centerSelectionMask):
    pocketCOMdata=pt.calc_center_of_mass(
        traj,pocketCOMmask)
    pocketCOMtable=pd.DataFrame({
        'Frame':np.arange(len(pocketCOMdata)),
        'X':pocketCOMdata[:,0],
        'Y':pocketCOMdata[:,1],
        'Z':pocketCOMdata[:,2]
    })
    return(pocketCOMtable)
def get_traj_pocket_resCOM_table(traj,residueSelectionMaskList,verbose=False):
    pocketResCOMtables=[]
    pocketResCOMs=np.array(
            [pt.calc_center_of_mass(tempTraj,residueSelectionMask) \
             for residueSelectionMask in residueSelectionMaskList])
    if verbose:
        frameIterator=tqdm.tqdm_notebook(
        np.arange(tempTraj.n_frames),desc='Compiling Pocket Residue COM table')
    else:
        frameIterator=np.arange(tempTraj.n_frames)
    for frameNum in frameIterator:
        pocketResCOMtables.append(pd.DataFrame({
```

```
            'Frame':[frameNum]*len(pocketResids),
            'ResidueMask':residueSelectionMaskList,
            'X':pocketResCOMs[:,frameNum,0],
            'Y':pocketResCOMs[:,frameNum,1],
            'Z':pocketResCOMs[:,frameNum,2]
        }))
    pocketResCOMtable=pd.concat(pocketResCOMtables)
    return(pocketResCOMtable)
def point_in_hull(point,hull,verbose=False):
    pointCoefs=np.array(
        [np.sum(hullFacet[:3] * \
                point) + \
         hullFacet[-1] \
         for hullFacet in hull.equations])
    if verbose:
        print(pointCoefs)
    return(np.sum(pointCoefs>0)<=0)
def points_in_hull(pointsArr,hull,verbose=False):
    #pointsArr= Npoints x 3 array
    pointsCoeffs=np.array(np.matrix(pointsArr)*np.matrix(hull.equations[:,:3].T)) + \
        hull.equations[:,3:4].T
    if verbose:
        print(pointsCoeffs)
    return(np.sum(pointsCoeffs>0,axis=1)<=0)
def get_hull_occupancy_mask(volGrid,hull,verbose=False):
    gridX,gridY,gridZ=np.meshgrid(
        volGrid.midpoints[1],
        volGrid.midpoints[0],
        volGrid.midpoints[2]
    )
    nzInds=np.nonzero(np.zeros(gridX.shape)+1)
    coordsArray=np.array([
        gridY[nzInds],gridX[nzInds],gridZ[nzInds]
    ]).T
    #if verbose:
        #crdArray=tqdm.tqdm_notebook(coordsArray)
    #else:
        #crdArray=coordsArray
    display(coordsArray[223561:223565])
    gridX=[]
    gridY=[]
    gridZ=[]
    gc.collect()
    outGrid=copy.deepcopy(volGrid)
    display(points_in_hull(coordsArray[223561:223565],hull,verbose=True))
    outGrid.grid[nzInds]=points_in_hull(coordsArray,hull,verbose=verbose)
    return(outGrid)
def get_pocket_ball_volume_mask(
    volGrid,
    pocketCenterCoords,
    pocketCenterRadius,
```

```
    pocketResidueCoords,
    pocketResidueRadii,
    returnPassCount=False):

    maskVolGrid=copy.deepcopy(volGrid)
    resBallMask=copy.deepcopy(volGrid)
    resBallMask.grid*=0
    gridY,gridX,gridZ=np.meshgrid(volGrid.midpoints[1],
                                  volGrid.midpoints[0],
                                  volGrid.midpoints[2])


    maskVolGrid.grid=1.*(np.sqrt((gridX-pocketCenterCoords[0])**2 + \
        (gridY-pocketCenterCoords[1])**2 + \
        (gridZ-pocketCenterCoords[2])**2)<=pocketCenterRadius)

    for iRes,resCoords in enumerate(pocketResidueCoords):
        resBallMask.grid+=1.*(np.sqrt((gridX-resCoords[0])**2 + \
            (gridY-resCoords[1])**2 + \
            (gridZ-resCoords[2])**2)<=pocketResidueRadii[iRes])
        #print(np.sum(resBallMask.grid))
    if not returnPassCount:
        resBallMask.grid=resBallMask.grid>0

    maskVolGrid.grid*=resBallMask.grid

    return(maskVolGrid)
def pathing_bfs(pathingGrid,startQueue=None,callback=None,verbose=False):
    #setup distance grid
    if verbose:
        print("setting up distance grid")
    distGrid=np.zeros(shape=pathingGrid.shape)
    pathableInds=np.nonzero(pathingGrid)
    distGrid=distGrid-1
    distGrid[pathableInds]=0
    gc.collect()

    maxDist=np.product(distGrid.shape)*2

    if verbose:
        print("initializing search queue")
    #Build search Queue from indices of pathable
    #grid nodes
    if startQueue is None:
        searchQueue=np.array([
            pathIndArr for pathIndArr in pathableInds
        ]).T
    else:
        searchQueue=startQueue

    if verbose:
```

```
        print("building search direction list")
    #setup search directions array
    Sdeltas=[-1,0,1]
    dirList=[Sdeltas]*searchQueue.shape[1]
    searchList=[
        sVec for sVec in itertools.product(*dirList) \
        if np.sum(np.abs(sVec))==1]
    if verbose:
        print('search directions:',searchList)
    if verbose:
        print("running bfs algorithm")
        print("queue:",searchQueue)
    while len(searchQueue)>0:
        #pop the top element of searchQueue
        searchInds=searchQueue[0,:]
        searchQueue=np.delete(searchQueue,0,0)
        distVal=distGrid[tuple(searchInds)]
        lastCallTime=int(round(time.time() * 1000))
        if distVal==0:
            distGrid[tuple(searchInds)]=1

            for searchDir in searchList:
                searchLoc=tuple(np.array(searchInds)+np.array(searchDir))
                searchVal=distGrid[searchLoc]
                if not callback is None:
                    currentTime=int(round(time.time() * 1000))
                    if (lastCallTime-currentTime) > 10:
                        callback(searchInds,distVal,searchLoc,searchVal)
                        lastCallTime=int(round(time.time() * 1000))
                if searchVal==0:
                    searchQueue=np.append(searchQueue,[list(searchLoc)],axis=0)

    return(distGrid)
def get_nearest_gridIndex(gridVol,coords):
    if not (len(coords)==len(gridVol.grid.shape)):
        raise ValueError(
            'Number of grid dimensions (%s) does not match number of coordinate dimensions
(%s)'%(
                len(gridVol.grid.shape),len(coords)))
    else:
        gridInds=(coords-gridVol.origin)/gridVol.delta
        if np.any(gridInds>np.array(gridVol.grid.shape)) or np.any(gridInds<0):
                raise Warning('coordinate grid indices %s out of bounds'%str(gridInds))
        return(gridInds)
def render_plotly_structure_lineplot(
    tempTraj,frameNum=0,renderFig=None,
    selectionMask='@CA',
    showRender=True,returnFig=False):

    bbInds=tempTraj.topology.atom_indices(selectionMask)
    bbCoords=tempTraj[frameNum].xyz[bbInds,:]
```

```
    if renderFig is None:
        fig=go.Figure()
    else:
        fig=renderFig
    fig.add_trace(
        go.Scatter3d(
            x=bbCoords[:,0],y=bbCoords[:,1],z=bbCoords[:,2],
            mode='lines'
        )
    )
    if showRender:
        fig.show()
    if returnFig:
        return(fig)

def render_plotly_volume_mask(
    volGrid,isoMin=0,isoMax=1,nSurf=1,renderFig=None,
    showRender=True,returnFig=False):
    Y,X,Z=np.meshgrid(volGrid.midpoints[1],
                      volGrid.midpoints[0],
                      volGrid.midpoints[2])
    if renderFig is None:
        fig=go.Figure()
    else:
        fig=renderFig
    fig.add_trace(
        go.Volume(
        x=X.flatten(),
        y=Y.flatten(),
        z=Z.flatten(),
        value=volGrid.grid.flatten(),
        isomin=isoMin,
        isomax=isoMax,
        opacityscale='max',
        opacity=0.75, # needs to be small to see through all surfaces
        surface_count=nSurf, # needs to be a large number for good volume rendering
        ))
    if showRender:
        fig.show()
    if returnFig:
        return(fig)

def render_plotly_CA_lineplot(
    tempTraj,frameNum=0,renderFig=None,
    showRender=True,returnFig=False):

    bbInds=tempTraj.topology.atom_indices('@CA')
    bbCoords=tempTraj[frameNum].xyz[bbInds,:]
    if renderFig is None:
        fig=go.Figure()
    else:
```

```
    fig=renderFig
  fig.add_trace(
    go.Scatter3d(
      x=bbCoords[:,0],y=bbCoords[:,1],z=bbCoords[:,2],
      mode='lines'
    )
  )
  if showRender:
    fig.show()
  if returnFig:
    return(fig)
```

CELL BREAK

Load trajectory

CELL BREAK

```
tempTraj=pt.iterload('autoimage_rep1.nc',
            top='step5_charmm2amber.parm7')
tempTraj
```

Note: Can also load a PDB file and parm7

CELL BREAK

```
tempTraj.topology.residue(400).original_resid
```

CELL BREAK

```
membResids=[res.original_resid for res in tempTraj.topology.residues \
 if ('POPC' in res.name) or ('CHL' in res.name)]
np.min(membResids),np.max(membResids)
```

Note: The lipids were used to prevent the breadth-width search from escaping the receptor laterally

CELL BREAK

Define pocket residues and center and build corresponding data tables

CELL BREAK

```
PocketResids="256 199 284 167 109 88 264 92 292"
pocketResids=np.array(list(map(int,pocketResids.split())),dtype=int)
print('pocket resids:',pocketResids)
pocketCOMmask=':'+','.join(list(map(str,pocketResids)))

#pocketResMaskList=[':%g'%resid for resid in pocketResids]

pocketResMaskList=[':256@HE1',':199@HZ2',':284@CE',':167@NH2',':109@OG',':88@HG1','
:264@CG2',':92@HE1',':292@HH']
```

Note: Can use center of mass instead of atoms with the pounded out line

CELL BREAK

```
pocketResCOMtable=get_traj_pocket_resCOM_table(tempTraj,pocketResMaskList)
```

```
pocketResCOMtable['Resid']=pocketResCOMtable['ResidueMask'].map(
    lambda x: int(x.replace(':','').split('@')[0]))

#pocketResCOMtable=pocketResCOMtable.rename(columns={'ResidueMask':'Resid'})
display(pocketResCOMtable.head())

pocketCOMtable=get_traj_pocket_centerCOM_table(tempTraj,pocketCOMmask)
pocketCOMtable.head()
```

CELL BREAK

> Note: Include protein and lipid, and can alter the densityCut if needed.
>
> This section creates a visualization of the system to ensure that the pocket is in the correct space.

```
proteinSelectionMask=':1-516'
gridDeltas=(1.,1.,1.)
densityCut=.001

@interact_manual
def render_traj_frame_pocket(
    frameNumber=widgets.IntSlider(
        min=pocketCOMtable.Frame.min(),
        max=pocketCOMtable.Frame.max()),
    residueBallRadius=widgets.FloatSlider(min=1,max=16,value=8.0)):

    print('Computing Protein Volume Map')
    tempVol=pt.all_actions.volmap(
        tempTraj[frameNumber:(frameNumber+1)],
        mask=proteinSelectionMask,
        grid_spacing=gridDeltas,buffer=2.0,
        centermask=proteinSelectionMask)
    tempVolDeltas=gridDeltas
    tempVolCenter=pt.center_of_mass(
        tempTraj[frameNumber:(frameNumber+1)],
        mask=proteinSelectionMask)[0]
    tempVolOrigin=tempVolCenter-np.array(list(tempVolDeltas)) * \
        (np.array(list(tempVol.shape),dtype=float)+5*np.array([1,1,-1]))/2.
    tempVolGrid=gridData.Grid(
        grid=tempVol,
        origin=tempVolOrigin,
        delta=tempVolDeltas)

    print('Constructing Pocket Mask')
    tableQueryStr='Frame == %g'%frameNumber
    pocketCenter=pocketCOMtable.query(tableQueryStr)[['X','Y','Z']].iloc[0].to_numpy()
    pocketResidueCoords=pocketResCOMtable.query(tableQueryStr)[['X','Y','Z']].to_numpy()
    pocketRadius=np.sqrt(miniball.get_bounding_ball(pocketResidueCoords)[1])

    pocketMask=get_pocket_ball_volume_mask(
        tempVolGrid,pocketCenter,pocketRadius,
        pocketResidueCoords,np.array([residueBallRadius]*len(pocketResidueCoords)),
        returnPassCount=True)
    solVol=copy.deepcopy(tempVolGrid)
    solVol.grid=solVol.grid<densityCut
```

```
    volGrid=pocketMask*solVol

    print('computing pathing')
    centerInds=get_nearest_gridIndex(tempVolGrid,pocketCenter)
    with tqdm.tqdm_notebook(desc='Computing Pathing') as pbar:
        def updateFun(inds1,dist1,inds2,dist2):
            pbar.set_description_str(str(inds1)+':'+str(dist1)+', ' +\
                                     str(inds2)+':'+str(dist2))
            pbar.update()
        pathVol=pathing_bfs(pathingGrid=volGrid.grid,
                  startQueue=np.array([centerInds],dtype=int),
                  #callback=updateFun,
                        verbose=False)

    volGrid.grid=volGrid.grid*(pathVol>0)

    print("Rendering")
    renderFig=render_plotly_CA_lineplot(
        tempTraj,frameNum=frameNumber,showRender=False,returnFig=True)
    renderFig.add_trace(
        go.Scatter3d(
            x=pocketResidueCoords[:,0],
            y=pocketResidueCoords[:,1],
            z=pocketResidueCoords[:,2],
            mode='markers'))
    render_plotly_volume_mask(volGrid,
                          isoMin=1,isoMax=np.max(volGrid.grid),
                          nSurf=int(np.max(volGrid.grid)+1),
                          renderFig=renderFig)
    display(pd.DataFrame({
        'Pocket_Contact_Threshold':np.arange(1,np.max(volGrid.grid)+1),
        'Voxel_Count_Volume':[
            np.sum(volGrid.grid >= cCut)
            for cCut in np.arange(1,np.max(volGrid.grid)+1)
        ]
    }))

CELL BREAK

#This may, apparently take quite a bit of memory to run
#and seems to have problems with some frames. If it fails,
#you can update the line 'np.arange(...,tempTraj.n_frames)'
#to restart from the last frame it got to (see the progress bar)
#it will save each frame on the fly, so you can load them after if needed
startFrame=0
proteinSelectionMask=':1-516'
gridDeltas=(1.,1.,1.)
pocketVolumeDataFrames=[]
densityCut=.001
residueBallRadius=8.0
with tqdm.tqdm_notebook(
```

Note: This current iteration has a memory leak and will crash at approximately 500 frames depending on the size. The start frame can be altered to the point of the crash to restart the breadth-width search

Be sure to update the proteinSelectionMask

```
    np.arange(startFrame,tempTraj.n_frames)
) as pbar:
    with tqdm.tqdm_notebook(desc='Computing Pathing') as pbar2:
        def updateFun(inds1,dist1,inds2,dist2):
            pbar2.set_description_str(str(inds1)+':'+str(dist1)+', ' +\
                                str(inds2)+':'+str(dist2))
            pbar2.update()
        for frameNumber in pbar:
            pbar.set_description_str('%50s'%('Computing Frame %g Volmap'%frameNumber))
            tempVol=pt.all_actions.volmap(
                tempTraj[frameNumber:(frameNumber+1)],
                mask=proteinSelectionMask,
                grid_spacing=gridDeltas,buffer=2.0,
                centermask=proteinSelectionMask)
            tempVolDeltas=gridDeltas
            tempVolCenter=pt.center_of_mass(
                tempTraj[frameNumber:(frameNumber+1)],
                mask=proteinSelectionMask)[0]
            tempVolOrigin=tempVolCenter-np.array(list(tempVolDeltas)) * \
                (np.array(list(tempVol.shape),dtype=float)+5*np.array([1,1,-1]))/2.
            tempVolGrid=gridData.Grid(
                grid=tempVol,
                origin=tempVolOrigin,
                delta=tempVolDeltas)

            pbar.set_description_str('%50s'%('Constructing Frame %g Mask'%frameNumber))
            tableQueryStr='Frame == %g'%frameNumber
            pocketCenter=np.array(pocketCOMtable.query(tableQueryStr)[['X','Y','Z']].iloc[0])
            pocketResidueCoords=np.array(pocketResCOMtable.query(tableQueryStr)[['X','Y','Z']])
            pocketRadius=np.sqrt(miniball.get_bounding_ball(pocketResidueCoords)[1])

            pocketMask=get_pocket_ball_volume_mask(
                tempVolGrid,pocketCenter,pocketRadius,
                pocketResidueCoords,
                np.array([residueBallRadius]*len(pocketResidueCoords)), #contact radii
                returnPassCount=True)

            solVol=copy.deepcopy(tempVolGrid)
            solVol.grid=solVol.grid<densityCut
            volGrid=pocketMask*solVol

            centerInds=get_nearest_gridIndex(tempVolGrid,pocketCenter)
            pathVol=pathing_bfs(pathingGrid=volGrid.grid,
                        startQueue=np.array([centerInds],dtype=int),
                        callback=updateFun,verbose=False)

            volGrid.grid=volGrid.grid*(pathVol>0)

            pbar.set_description_str('%50s'%('Compiling Frame %g Volume Data'%frameNumber))
            pocketVolumeDataFrames.append(pd.DataFrame({
                'Frame':[frameNumber]*len(np.arange(1,np.max(volGrid.grid)+1)),
```

```
            'Pocket_Contact_Threshold':np.arange(1,np.max(volGrid.grid)+1),
            'Voxel_Count_Volume':[
                np.sum(volGrid.grid >= cCut)
                for cCut in np.arange(1,np.max(volGrid.grid)+1)
            ]
        }))
        pbar.set_description_str('saving frame %g data'%frameNumber)
        pocketVolumeDataFrames[-
1].to_csv('pocketVolumeData.Frame_%g.csv'%frameNumber)
        #else:
        #    print("Warning: Pocket COM is occluded in frame %g"%frameNumber)
        gc.collect()
        pbar2.reset()
pocketVolumeDataFrame=pd.concat(pocketVolumeDataFrames)
pocketVolumeDataFrame.head()
```

CELL BREAK

```
volGrid.grid[tuple(np.array(centerInds,dtype=int))]
```

CELL BREAK

```
pocketResCOMtable.head(n=8)
```

CELL BREAK

```
volDataFiles=[filename for filename in os.listdir('./') \
              if ('pocketVolumeData' in filename) and ('Frame_' in filename)]

pocketVolumeDataFrames=[]
for filename in volDataFiles:
    pocketVolumeDataFrames.append(pd.read_csv(filename))
pocketVolumeDataFrame=pd.concat(pocketVolumeDataFrames)
pocketVolumeDataFrames=[]
pocketVolumeDataFrame=pocketVolumeDataFrame[
    [colname for colname in pocketVolumeDataFrame.columns \
             if not ('Unnamed' in colname)]]
gc.collect()
pocketVolumeDataFrame.head()
```

CELL BREAK

```
fig=go.Figure()
plotData=pocketVolumeDataFrame.query(
    'Pocket_Contact_Threshold == 1.0'
).sort_values(['Frame','Pocket_Contact_Threshold'])
fig.add_trace(
    go.Line(x=plotData.Frame,
            y=plotData['Voxel_Count_Volume']))
fig.show()
```

Note: Will create a visualization of the data

CELL BREAK

```
plotData.to_csv('Pocket_Volume_Plot_Data.csv',index=None)
```
