# Supplementary material for: In silico prediction of ARB resistance: A first step in creating personalized ARB therapy
Source: PLoS Comput Biol. 2020 Nov 25;16(11):e1007719. doi: 10.1371/journal.pcbi.1007719 (PMC7725353; doi:10.1371/journal.pcbi.1007719)
Supplement: Attachment — Submitted filename: ref_report.docx [file pcbi.1007719.s008.docx]

Anderson et al presents the manuscript ‘In silico prediction of ARB resistance: A first step in creating personalized ARB therapy” where they performed molecular dynamics of the Angiotensin II type 1 receptor followed by docking of 8 known antagonists. The authors describe a potential method for personalized treatment of patients taking single nucleotide polymorphism into account. The paper presents a fast, cheap and interesting approach for personalized treatment, but leaves a bit too much to the readers imagination, see comments below. Most of the comments regards further explanation to what has been done in the present study as I find some parts to be cut a bit short.

My main concerns regard the conclusion that AutoDock default parameters are the reason why experimentally determined binding affinities cannot be reproduced, and, the structural analysis of the simulations that determines which conformation to use throughout this study seems poor.

Comments:

The introduction seems to be on rather short, just about 400 words. Please expand this part.

*Page 5. “Herein, an empty AT1R was generated through molecular dynamic (MD) simulation, 103 nsSNPs from the 1000 genomes project were briefly modeled”*

What does it mean that the empty AT1R was generated through MD? I assume it was taken from an experimentally solved structure, missing residues were modelled, and the final model was subjected to further refinement using an MD method?

*Page 5. “A single frame representing the average stable root-mean-square deviation (RMSD) from the last 20 ns of replica 1 was chosen as the representative model of the empty AT1R; the PDB file format is available in the supplemental files.”*

On which criterions, besides a stable rmsd, were taken into account for this single frame to represent an entire ensemble of conformations generated through the MD? Was the rmsd calculated for all atoms or only backbone? Looking at the rmsd evolution in figure S1A it appears that the 3 replicated simulations are different, not much, but enough to think there could have been some sort of cluster analysis done to get a statistically more representative conformation.

Please explain in more detail how you chose this frame as your model as it is not entirely clear to me.

*Page 6. “…which Wingler et al. call TM1-TM2, and D236-R311, called TM6-helix 8…”*

Please add reference here to Wingler et al.

*Page 6. “…especially since the AT1R is known to display basal activity.”*

If it is known, please add reference to that fact.

*Page 7. “Therefore, the empty AT1R model is a model of the inactive human AT1R.”*

There are two crystal structures available for AT1R, 4ZUD and 4YAY. How does your model compare with 4YAY since, if I understand it, 4YAY was used as template for modelling of missing residues?

*Page 7. “The AutoDock predicted affinity for four ARBs (eprosartan, irbesartan, olmesartan, and telmisartan) was below the lowest reported experimentally derived affinity, and three ARBs (azilsartan, candesartan, and EXP3174, the active metabolite of losartan) were outside of 50% of all reported affinities.”*

If the docking pose matched the binding site but did not generate the correct biding affinities, why jump to the conclusions that there is something wrong with the docking parameters and not the structure on which the docking was done? Did you try any other docking software to check what caused the affinities to be different? For instance, Autodock Vina (which is open source), or docking with MOE. Or, did you check the ranking of the docked affinities and the experimentally determined ones to see if they matched?

*Page 8. “The mean of all the optimized parameters fell within the reported range of the affinity for the respective ARB.”*

The mean of the optimized docking parameters or do you mean the mean predicted affinities using the optimized parameters? The phrasing is a bit confusing.

*Page 10. “…*unpublished homology modeling followed by MD simulation of the AT1R from our group…”

I suggest removing this sentence as it does not add anything.

*Page 11. “*AutoDock was chosen specifically for the ability to obtain an estimated affinity, and as shown in Figure 4, the original parameters did not produce affinities within the known range for olmesartan although the orientation was accurate (not shown)”

See comment about using other softwares to verify that AutoDock default docking parameters are “bad”. You have conducted a large-scale docking study but choose to not show or compare the generated docking poses between the default docking parameters and the optimized parameters. Why not?

Page 14. Please add reference to the membrane builder of charmm-gui.

*Page 14. “The frame representing the value closest to the average RMSD of the stable 20 ns from replica 1 was selected as the structure representing the empty conformation of the AT1R and herein is called the empty AT1R model.”*

Goes back to my comment above, how was the rmsd determined? As the rmsd figure show, the structures may have reached some sort of energy minimum, but have not converged to the same minimum, e.g. the same rmsd, thus choosing the frame with lowest rmsd does not seem fitting.

If alpha carbons are the determining factor, were all residues included or only residues part of the TM helices? Please explain.

*Page 14. “based on the identified interactions within the crystal structures, the atoms nearest each other were identified and the distances measured in..”*

The standard is to measure distances between heavy atoms, how was it performed here?

*Page 15. “…and docking was conducted as described by S. Forli et al.(25).”*

Since S. Forli is not part of this paper, explain in detail how the docking was performed.

*Page 15. “…and RT is…”*

Please treat R and T separate in the text. The MD simulations were performed at 310K, was the docking done so too? Would it matter if the simulation and the docking was performed at different temperatures?

*Page 16. “After changing the residue, an energy minimization utilizing Amber10 EHT force field was conducted via MOE to generate an appropriate conformation of the AT1R carrying the polymorphism.”*

Why not simulate the mutated structure to see how it would affect the overall conformation? As you performed 150 ns of simulation to justify the selection of the structure to use, a similar simulation could have been done, as the length of these simulations are now achievable in less than 24 hours.

During minimization in MOE, was there bb restraints used only letting the side chains adopt to the newly introduced residue?

Figures should be referenced as Fig in the main text.

Figure 2. The double notation of residues in the figure is a bit confusing. I suggest only labeling the residue at each residue, and remove the top labels, similarly as in figure 3.

The position of the residues is also a bit hard to see due to the ball and stick representation. I suggest showing them as sticks (perhaps thinner sticks) and remove hydrogens.

Figure 3B does not really add anything in the current form. I suggest showing the difference in binding site volume differently, e.g. if helices are moving tighter in the apo form have an overlay of helices.

Correct the reference to figures to be of the correct standard.
