# Supplementary material for: In silico prediction of ARB resistance: A first step in creating personalized ARB therapy
Source: PLoS Comput Biol. 2020 Nov 25;16(11):e1007719. doi: 10.1371/journal.pcbi.1007719 (PMC7725353; doi:10.1371/journal.pcbi.1007719)
Supplement: Attachment — Submitted filename: Reply to Reviewers.docx [file pcbi.1007719.s009.docx]

**Reply to the reviewers**

Thank you for the thoughtful and helpful reviews. I believe that the changes made to the manuscript based on your review has improved the manuscript. Before answering each point, I would like to state that this project has been very slow moving; it began before any AT_1_R was crystalized and had to be completely rebooted after the first crystal structure was published. Now while trying to publish the work we have three crystal structures, and many authors as they have all been part of this project. I am revising the manuscript to contain data about the new active AT_1_R crystal and addressing, to the best of my ability, your critiques. Our specific comments to your critiques are provided below in red text. The manuscript is extensively modified based on your comments and a desire to more fully explore the apo-AT_1_R structure presented in the manuscript. We believe that the manuscript is much improved in great part due to your critical review of the document. Thank you.

Reply to Reviewer 1:

1)	Authors stated that they used 4ZUD pdb coded AT1R and they stated that "Specifically, the N-Terminus (residues 1 to 25), intracellular loop 2 (residues 134 to 140),extracellular loop 2 (residues 186 to 188), intracellular loop 3 (residues 223 to 234), and helix 8 (residues 305 to 316) were added to the AT1R in accordance to the human AT1R sequence and PDB:4YAY." It is not clear, why authors did not use 4YAY pdb coded AT1R directly? 4YAY also have a selective inhibitor at the binding pocket.

As stated in the preamble, this project began before there was a crystal structure of the AT_1_R. The project had to be rebooted when the crystal structures demonstrated that R167 is pointed into the binding pocket. This orientation provides an additional positive charge in the binding pocket, which clearly will alter the binding of dianionic and monoanionic ARBs. All homology models (published and our own) had R167 pointing away from the binding pocket and towards the solvent. Thus, we switched to 4ZUD as soon as we learned about the structure as it has Olmesartan bound, which is a clinically prescribed ARB and one that was of interest due to our previous work (PMID: 23389452). Additionally, 4ZUD is slightly higher resolution than 4YAY (2.8 Å versus 2.9 Å). We hope this history explains our choices. The silver lining to this choice is that, as per point two below, we can compare our structure directly to 4YAY.

2)	Authors mentioned that "Despite the difference in the distances, structural alignment of the DRY, PIF, and NPxxY motifs between the empty AT1R model and inactive A2AR crystal structure (PDB:3EML) demonstrate that the empty AT1R model is inactive (Figure 2). The NPxxY motif is not as clearly aligned as the DRY and PIF, but this is primarily due to the unique position of helix 8 in the empty AT1R model. For further comparison, the empty AT1R model was aligned to the active mu-opioid receptor (PDB:5C1M), demonstrating the drastic shifts in the DRY, PIF, and NPxxY required to assume the active state." It is not clear why the authors did not use directly active and inactive states from available angiotensin receptors? (i.e. 6do1)

You make an excellent point. The rationale for not using the AT_1_R crystal structures is the history of this project, but given that the PDBs are available, our model should be aligned to the inactive AT_1_R 4YAY and the active AT_1_R 6DO1. Thus, we have altered the manuscript to use the inactive and active AT_1_R crystal structures in Figure 2 and Supplemental Figure 2B, respectively. However, as you will see in the figures, the active AT_1_R does not have an altered DRY motif; thus, we are keeping the old figures to help confirm that the model is inactive (A2aR 3EML) and not active (mOR 5C1M). These figures are now Supplemental Figures 2A and 2C, respectively.

3)	It is well known that GPCRs may exist and function as monomers; however, they can assemble to form higher order structures, and as a result of oligomerization, their function and signaling profiles can be altered. Recent findings reveal that AT1R can form homodimers and activate the noncanonical (β-arrestin-mediated) pathway. Thus, effect of dimerization to the binding pocket should not be overlooked.

Again, you make a good point. We have mentioned this in the discussion, where we discuss the limitations of this study. However, the effect of dimerization is beyond the scope of our current study.

4)	It is stated that "Although there is little experimental data for direct comparison, the docking correctly predicted the direction of the change in affinity 60% of the time and was within two times the calculated error 60% of the time" It is not clear how this ratio is found?

We apologize for the confusion. We have removed this statement and just indicated how we compare to the work by Arsenault J. et al.

5)	In Figure 3, instead showing the volume change with single snapshot, authors may consider to show volume change during the simulations.

Another good point, we created a new Figure 3B describing the dynamic collapse of the pocket after the removal of the ligand. We also conducted further analysis on the model AT_1_R to determine if it is a valid model of the inactive apo-AT_1_R by looking at water and sodium within the ligand-binding pocket.

6)	A better representation of the results is needed. Provided details in discussion and results are not enough.

We have extensively modified the results, added new figures and tables, and expanded the methods.

Minor points:

There are 15 authors for this full computational study. It would be good to add a section showing each author's contribution to the paper.

We have added our already prepared author roles to the manuscript; I inaccurately assumed that it would be part of submission. It is now at the end of the manuscript.

**Reviewer 2**

Anderson et al presents the manuscript ‘In silico prediction of ARB resistance: A first step in creating personalized ARB therapy” where they performed molecular dynamics of the Angiotensin II type 1 receptor followed by docking of 8 known antagonists. The authors describe a potential method for personalized treatment of patients taking single nucleotide polymorphism into account. The paper presents a fast, cheap and interesting approach for personalized treatment, but leaves a bit too much to the readers imagination, see comments below. Most of the comments regards further explanation to what has been done in the present study as I find some parts to be cut a bit short.

My main concerns regard the conclusion that AutoDock default parameters are the reason why experimentally determined binding affinities cannot be reproduced, and, the structural analysis of the simulations that determines which conformation to use throughout this study seems poor.

Comments:

The introduction seems to be on rather short, just about 400 words. Please expand this part.

*Page 5. “Herein, an empty AT1R was generated through molecular dynamic (MD) simulation, 103 nsSNPs from the 1000 genomes project were briefly modeled”*

What does it mean that the empty AT1R was generated through MD? I assume it was taken from an experimentally solved structure, missing residues were modelled, and the final model was subjected to further refinement using an MD method?

Correct assumption. We have all of that information in the methods and results, but we added a brief description to the introduction, as you instructed. The generation is part of the research that took many years as we started with homology models. While looking to publish the manuscript, the crystal structure was published with Arg 167 pointed towards that binding pocket, which is not what any homology-based model predicted. Thus, we were forced to start from the beginning of the project utilizing the crystal structure containing Olmesartan (4ZUD) as our experimentally solved structure.

*Page 5. “A single frame representing the average stable root-mean-square deviation (RMSD) from the last 20 ns of replica 1 was chosen as the representative model of the empty AT1R; the PDB file format is available in the supplemental files.”*

On which criterions, besides a stable rmsd, were taken into account for this single frame to represent an entire ensemble of conformations generated through the MD? Was the rmsd calculated for all atoms or only backbone? Looking at the rmsd evolution in figure S1A it appears that the 3 replicated simulations are different, not much, but enough to think there could have been some sort of cluster analysis done to get a statistically more representative conformation.

Please explain in more detail how you chose this frame as your model as it is not entirely clear to me.

In traditional docking projects, ensemble docking with multiple receptor conformations extracted from clustering analysis has been shown to improve the prediction power. However, the current study alters the paradigm of traditional docking. Instead of docking a large number of small molecules into a handful of various states of a single receptor, here we are dealing with a large number of receptors and a handful of ligands (103 known polymorphisms plus the wild-type receptor are docked to 8 different drugs [832 different systems], and each system is run through 100 docking poses six times). Therefore, ensemble docking is not feasible at the current stage. Instead of just arbitrarily picking the last frame, we argue that the frame with protein backbone RMSD closest to the mean stable RMSD would be a better representative of the ensemble average than the last frame. Additionally, using the average RMSD provided a mathematical guide to “blindly” choose a single frame independent of its structure to move onward to mutagenesis studies. It should be noted that this process is what produced a structure where helix VIII is congruent with Helix VII, which was a surprise and would not have been chosen in a non-blinded fashion. All the data regarding the receptor movement and structure are based on the ensemble of stable trajectories. We will clarify this in the manuscript, as well as clarify the limitations.

*Page 6. “…which Wingler et al. call TM1-TM2, and D236-R311, called TM6-helix 8…”*

Please add reference here to Wingler et al.

Done

*Page 6. “…especially since the AT1R is known to display basal activity.”*

If it is known, please add reference to that fact.

Done

*Page 7. “Therefore, the empty AT1R model is a model of the inactive human AT1R.”*

There are two crystal structures available for AT1R, 4ZUD and 4YAY. How does your model compare with 4YAY since, if I understand it, 4YAY was used as template for modelling of missing residues?

The same point was brought up by reviewer number 1, and it is an excellent point that improves the manuscript substantially. The following is copied from our response to reviewer 1.

You make an excellent point. The rationale for not using the AT_1_R crystal structures is the history of this project, but given that the PDBs are available, our model should be aligned to the inactive AT_1_R 4YAY and the active AT_1_R 6DO1. Thus, we have altered the manuscript to use the inactive and active AT_1_R crystal structures in Figure 2 and Supplemental Figure 2B, respectively. However, as you will see in the figures, the active AT_1_R does not have an altered DRY motif; thus, we are keeping the old figures to help confirm that the model is inactive (A2aR 3EML) and not active (mOR 5C1M). These figures are now Supplemental Figures 2A and 2C, respectively.

*Page 7. “The AutoDock predicted affinity for four ARBs (eprosartan, irbesartan, olmesartan, and telmisartan) was below the lowest reported experimentally derived affinity, and three ARBs (azilsartan, candesartan, and EXP3174, the active metabolite of losartan) were outside of 50% of all reported affinities.”*

If the docking pose matched the binding site but did not generate the correct biding affinities, why jump to the conclusions that there is something wrong with the docking parameters and not the structure on which the docking was done? Did you try any other docking software to check what caused the affinities to be different? For instance, Autodock Vina (which is open source), or docking with MOE. Or, did you check the ranking of the docked affinities and the experimentally determined ones to see if they matched?

We initially ran docking in MOE as well, and have gone back to run the docking of Olmesartan in Autodock Vina, and MOE. We have created a figure showing the binding poses closest to the crystal structure in a new figure and provide the Boltzmann average binding affinity. Indeed, Vina does get one pose close to the crystal structure, but the affinity calculation is not correct. The most significant problem in docking ARBs is that the AutoDock software does not know how to properly apply charges to the tetrazole ring found on Olmesartan and most other ARBs; therefore, even with the proper pose, the affinity calculation is incorrect. We bring this up in the revised discussion. A previous student from our program is working on the parameterization of tetrazole rings for her thesis, which exemplifies that such parameterization is beyond the scope of this project.

*Page 8. “The mean of all the optimized parameters fell within the reported range of the affinity for the respective ARB.”*

The mean of the optimized docking parameters or do you mean the mean predicted affinities using the optimized parameters? The phrasing is a bit confusing.

Yes, your understanding is correct, and we have modified the sentence to make it clear.

*Page 10. “…*unpublished homology modeling followed by MD simulation of the AT1R from our group…”

I suggest removing this sentence as it does not add anything.

We have modified this sentence to read, “homology modeling failed to accurately predict the orientation of Arg167.” This statement is more direct and hopefully adds something to the conversation regarding the structure of the apo-AT_1_R.

*Page 11. “*AutoDock was chosen specifically for the ability to obtain an estimated affinity, and as shown in Figure 4, the original parameters did not produce affinities within the known range for olmesartan although the orientation was accurate (not shown)”

See comment about using other softwares to verify that AutoDock default docking parameters are “bad”. You have conducted a large-scale docking study but choose to not show or compare the generated docking poses between the default docking parameters and the optimized parameters. Why not?

Only a handful of the poses from each Autodock run match the crystal structure. No docking program resulted in all the poses matching the crystal orientation, including in Vina. MOE never obtained a pose resembling the crystal structure. We now provide a snippet of the poses that can be compared to the crystal structure and the energies, as described previously (Fig. 4). The original parameters were altered to get the affinity to match known wild-type values, the parameters altered just dealt with the search space, not he ligand or receptor files. We have changed how we discuss these parameters in the manuscript.

Page 14. Please add reference to the membrane builder of charmm-gui.

Done

*Page 14. “The frame representing the value closest to the average RMSD of the stable 20 ns from replica 1 was selected as the structure representing the empty conformation of the AT1R and herein is called the empty AT1R model.”*

Goes back to my comment above, how was the rmsd determined? As the rmsd figure show, the structures may have reached some sort of energy minimum, but have not converged to the same minimum, e.g. the same rmsd, thus choosing the frame with lowest rmsd does not seem fitting.

If alpha carbons are the determining factor, were all residues included or only residues part of the TM helices? Please explain.

We apologize for being too vague. The details of our RMSD calculation has been clarified in the manuscript. The RMSD was computed with the alpha carbons of the helixes. The N- and C-terminus plus loops were excluded from the RMSD calculation due to their significant fluctuation.

*Page 14. “based on the identified interactions within the crystal structures, the atoms nearest each other were identified and the distances measured in..”*

The standard is to measure distances between heavy atoms, how was it performed here?

The distance between the center of mass of amino acid was measured over the entire stable trajectory using CPPtraj. Measurements in the PDBs were remeasured using UCSF Chimera and were also set to the center of mass of each residue.

*Page 15. “…and docking was conducted as described by S. Forli et al.(25).”*

Since S. Forli is not part of this paper, explain in detail how the docking was performed.

Done

*Page 15. “…and RT is…”*

Please treat R and T separate in the text. The MD simulations were performed at 310K, was the docking done so too? Would it matter if the simulation and the docking was performed at different temperatures?

R and T are now separated as aked. When temperature could be altered, it was set to 310.15 K.

*Page 16. “After changing the residue, an energy minimization utilizing Amber10 EHT force field was conducted via MOE to generate an appropriate conformation of the AT1R carrying the polymorphism.”*

Why not simulate the mutated structure to see how it would affect the overall conformation? As you performed 150 ns of simulation to justify the selection of the structure to use, a similar simulation could have been done, as the length of these simulations are now achievable in less than 24 hours.

During minimization in MOE, was there bb restraints used only letting the side chains adopt to the newly introduced residue?

The MD time required to equilibrate fully after the polymorphism is introduced is extensive and polymorphism dependent. None of the polymorphisms examined to date are stable until 300 ns at the earliest. We began some of these studies but have not completed them to date. Additionally, given our current cluster and usage, I calculated that it would take at least 30 years to get a stable system for each polymorphism, which is beyond the scope of a paper. We have emphasized in the paper that ideally, each polymorphism should be run in MD until stable and then introduce the ARBs, and continue MD simulation with an ARB present. MM-GBSA should be run to determine a more accurate affinity for each ARB to each polymorphism. We have begun these studies with Eprosartan as it does not have the tetrazole ring found in Olmesartan. However, we have only completed the simulation for the wild-type AT_1_R.

During MOE minimization, there were no restraints on the protein. This is also why it was minimized in the membrane as minimization without a membrane (vacuum) distorts the receptor conformation. We have clarified this in the manuscript

Figures should be referenced as Fig in the main text.

Done

Figure 2. The double notation of residues in the figure is a bit confusing. I suggest only labeling the residue at each residue, and remove the top labels, similarly as in figure 3.

Done

The position of the residues is also a bit hard to see due to the ball and stick representation. I suggest showing them as sticks (perhaps thinner sticks) and remove hydrogens.

Done

Figure 3B does not really add anything in the current form. I suggest showing the difference in binding site volume differently, e.g. if helices are moving tighter in the apo form have an overlay of helices.

Agreed, this is of limited value as it is one slice and not dynamic. We have altered the figure so now we show a dynamic representation of the binding pocket over time.

Correct the reference to figures to be of the correct standard.

Done
